# Nitrate of Amyl and Nitro-glycerine in Uræmic Asthma

**Published:** 1883-09

**Authors:** 


NITRATE OF AMYL AND NITRO-GLYCERINE IN URÆMIC ASTHMA.—Dr. Sheen, of Cardiff, writes:

"The brief notes I give below illustrate the value of nitrite of amyl and nitro-glycerine in one of the sudden and distressing, though perhaps rare, phases of chronic Bright's disease—viz., uræmic asthma. Nitrite of amyl, acting, probably, through the vasomotor nerves, relaxes the arterioles, and thus reduces blood pressure. As it is very volatile, on the score of economy and convenience, I always carry some of Martindale's capsules in my bag, and these are very handy for immediate use. Nitroglycerine is said to have much the same action as nitrite of amyl, and, according to Dr. Mahomed, its great superiority over amyl lies in its gradual and more lasting effect, and the more convenient manner of prescribing it, and it can be taken regularly two or three times a day, or oftener, in one minim of a one-per-cent. alcoholic solution being the usual commencing dose. It is also made up in chocolate tablets, each containing one-hundredth part of a minim; but its action, when given in this form, is not so rapid as that of the alcoholic solution.

M. P., aged 55, retired from business May 4, 1882. Has been ailing for two weeks, but has been about. Has noticed swelling of legs toward night for two months, and his face has swollen occasionally for the last month. Has always been careless of his health, and if he got wet—an event which happened not unfrequently—he would never change his clothes. He was taken suddenly ill last evening while out walking, about a mile from home, and had to be taken home in a cab. On visiting him at 10 A.M., I found him sitting up in bed, gasping for breath, countenance distressed, and of a sickly, pallid hue. Pulse feeble; temperature 98°; tongue pale and sodden; expectoration frothy, with some little blood intermixed; moist *roles* over whole chest, back and front; urine abundant, clear, containing one-fourth of albumen. At 2 P.M. I found his condition and posture unchanged; he could only speak a few words before he had to stop for breath. He inhaled three minims of nitrite of amyl (a capsule broken in a handkerchief). Within a few minutes his breath was easier, and he was able to recline in bed for the first time since the attack came on before I left the house. I then put him on nitro-glycerine one hundredth part of a minim *ter die.*

May 5—He was lying easily in bed, breathing quietly, and expressing himself as feeling quite well, said he was only waiting till I came before he got up. I cautioned him that his life hung by a thread, and that he could only hope to continue it by the strictest obedience. Unavailing caution. On the 6th he still remained in the same improved condition. The next day he refused to take any more medicine, but promised to stay in the house—a promise which he did not keep. On the 16th he had another attack, and died quietly within thirty-six hours, the urine being loaded with albumen."—*British Med. Journal.*

